# Changes in metabolic syndrome and the risk of breast and endometrial cancer according to menopause in Korean women

**DOI:** 10.4178/epih.e2023049

**Published:** 2023-05-01

**Authors:** Thi Xuan Mai Tran, Soyeoun Kim, Boyoung Park

**Affiliations:** 1 Department of Preventive Medicine, Hanyang University College of Medicine, Seoul, Korea; 2 Institute for Health and Society, Hanyang University, Seoul, Korea; 3 Hanyang Institute of Bioscience and Biotechnology, Hanyang University, Seoul, Korea

**Keywords:** Metabolic syndrome, Breast neoplasms, Endometrial neoplasms, Obesity, Prevention, Early detection of cancer

## Abstract

**OBJECTIVES:**

This study investigated how changes in metabolic syndrome (MetS) are associated with the subsequent risk of breast and endometrial cancer according to menopausal status.

**METHODS:**

This cohort study, using data from the National Health Insurance Service database, included women aged ≥40 years who underwent 2 biennial cancer screenings (2009-2010 and 2011-2012) and were followed up until 2020. Participants were grouped into MetS-free, MetS-recovery, MetS-development, and MetS-persistent groups. Menopausal status (premenopausal, perimenopausal, and postmenopausal) was assessed at 2 screenings. Cox proportional hazard regression was used to assess the association between MetS changes and cancer risk.

**RESULTS:**

In 3,031,980 women, breast and endometrial cancers were detected in 39,184 and 4,298, respectively. Compared with the MetS-free group, those who recovered, developed, or had persistent MetS showed an increased risk of breast cancer, with adjusted hazard ratios (aHRs) of 1.05, 1.05, and 1.11, respectively (p<0.005). MetS persistence was associated with an increased risk of breast cancer in postmenopausal women (aHR, 1.12, 95% confidence interval [CI], 1.08 to 1.16) but not in premenopausal or perimenopausal women. MetS persistence was associated with an increased risk of endometrial cancer in premenopausal, perimenopausal, and postmenopausal women, with aHRs of 1.41 (95% CI, 1.17 to 1.70), 1.59 (95% CI, 1.19 to 2.12), and 1.47 (95% CI, 1.32 to 1.63), respectively.

**CONCLUSIONS:**

Increased breast cancer risk was associated with recovered, developed, and persistent MetS in postmenopausal women. Meanwhile, increased endometrial cancer risk was found in obese women who recovered from MetS or persistently had MetS, regardless of menopausal status, when compared to MetS-free women.

## GRAPHICAL ABSTRACT

[Fig f3-epih-45-e2023049]

## INTRODUCTION

Metabolic syndrome (MetS) is a growing global problem [1,2]. MetS comprises a cluster of risk factors, including central adiposity, impaired fasting plasma glucose (FPG), elevated blood pressure (BP), elevated triglyceride (TG) levels, and low high-density lipoprotein (HDL) cholesterol levels. Breast cancer is the most commonly diagnosed cancer worldwide, accounting for 11.7% of all new cancer cases in 2020 [3]. In Korea, the burden of breast cancer has gradually increased over the last decade [4,5]. While endometrial cancer is less common, its incidence also has increased steadily in Korea [6]. Despite a generally decreasing trend in the incidence of total cancer and most cancer types, breast and endometrial cancer have shown steadily increasing trends in Korea since 1999, when the first national cancer statistics were published [7]. MetS has been reported to be associated with an increased risk of various malignancies, including endometrial cancer [8,9] and breast cancer [10,11]. The biological mechanisms underlying the association between MetS and breast and endometrial cancer might reflect the role of MetS as a potential surrogate marker for obesity, an unhealthy diet, and the aging process [1,12].

Previous studies have found an increased risk of breast cancer in postmenopausal, but not premenopausal women with MetS, suggesting that the association between MetS and breast cancer might differ depending on menopausal status [11-13], although evidence in perimenopausal women has remained limited. In addition, the association between adiposity and risk of breast cancer has shown opposite directions in premenopausal and postmenopausal women; high body mass index (BMI) had no association or was associated with a reduced risk of breast cancer in premenopausal women [14], whereas increased adiposity was positively associated with an increased risk of breast cancer in postmenopausal women [15]. Thus, the relationship between MetS and the risk of breast cancer might vary according to menopausal status and should be considered together with BMI status. While an association between MetS and breast cancer was found only in postmenopausal women [11-13], an increased risk of endometrial cancer associated with MetS was found in both premenopausal and postmenopausal women [8,9,16], with an even higher risk in premenopausal women [16].

Despite the existence of comprehensive evidence suggesting the important role of MetS as a risk factor for breast and endometrial cancer in postmenopausal women, it is unclear how changes in MetS affect the risk of cancer. Thus, this study aimed to assess the associations between longitudinal changes in MetS and the risk of developing breast and endometrial cancer. Because menopause has been proposed as an important factor contributing to an increased prevalence of MetS [17], we assessed the associations between changes in MetS and cancer development separately for premenopausal, perimenopausal, and postmenopausal women.

## MATERIALS AND METHODS

### Study sample or population

**Figure 1.**
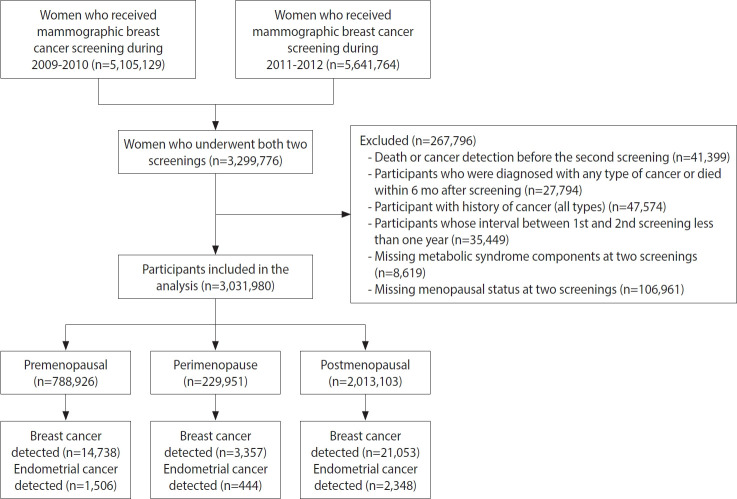
Flow diagram used to select the eligible population.

This study used customized data from the National Health Insurance Service (NHIS) database [18]. In Korea, the NHIS provides biennial health-screening examinations for all Koreans to assess the risk of chronic diseases. All inpatient and outpatient visits, procedures, and prescriptions are recorded in the database. The initial study database included women aged ≥ 40 years who underwent national breast cancer screening between 2009 and 2010 (n= 5,105,129) and between 2011 and 2012 (n= 5,641,764). Only participants who underwent screening during both periods were initially considered (n=3,299,776) (Figure 1). After excluding those who were diagnosed with cancer before the second screening or within 6 months after the second screening and those with missing information on MetS or missing menopausal status, the final dataset included 3,031,980 women.

### Measures of changes in metabolic syndrome

MetS was defined based on the modified National Cholesterol Rationale Education Program Adult Treatment Program (NECP-ATP III) [19], comprising the following 5 components: waist circumference (WC) ≥ 80 cm, FPG ≥ 100 mg/dL, TG level ≥ 150 mg/dL, HDL level < 50 mg/dL, and elevated BP (systolic ≥ 130 mmHg or diastolic ≥ 85 mmHg). The presence of each component of MetS was counted and added to obtain a score for each subject. An individual was defined as having MetS if he or she had 3 or more of the 5 above-mentioned components.

**Figure 2.**
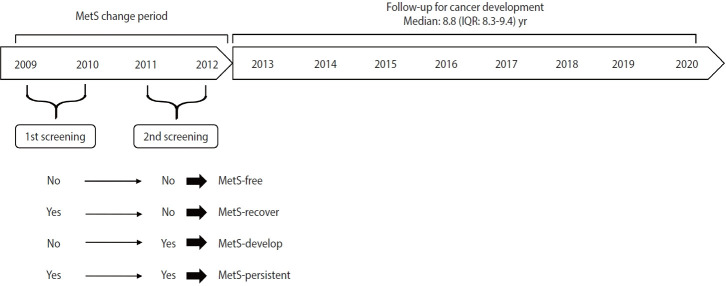
Flow diagram of the study design. MetS, metabolic syndrome; IQR, interquartile range.

According to changes in MetS status between the first screening in 2009-2010 and the second screening in 2011-2012, participants were grouped into the following categories: MetS-free (no MetS at both screenings), MetS-recovered (MetS at the first screening and no MetS at the second screening), MetS-developed (no MetS at the first screening and MetS at the second screening), and MetS-persistent (MetS at both screenings) (Figure 2).

In addition, changes in each component of MetS status between the first and second screenings were analyzed according to each menopausal group. Changes in MetS scores and the presence of each component of MetS between the first and second measurements and their associations with breast and endometrial cancer were assessed as described above.

### Measures of outcomes

The main outcomes were incident breast cancer and endometrial cancer, which were ascertained from the healthcare utilization database of the NHID. In Korea, patients with cancer have a special co-payment reduction program called the rare incurable disease system, and cancer cases are registered in this system from the time of diagnosis. In this study, the incidence of cancer events was defined as cases having both the corresponding International Classification of Diseases code (C50 or D05 for breast cancer and C54 for endometrial cancer) and a rare incurable disease system code [20]. This approach is sufficiently reliable and has been used in previous studies [21]. Cancer case tracking was conducted until December 2020. If a woman developed both breast cancer and endometrial cancer, the first cancer was considered an outcome.

### Measures of other covariates

Menopausal status was measured at both screenings using the question: “What is your current menopausal status?” with the following response options: still menstruating, have had a hysterectomy, and have undergone menopause. Women who reported having undergone menopause were further asked to report the age at which they underwent menopause. Based on this information, menopausal status was classified into the following categories: premenopausal, perimenopausal, and postmenopausal. Perimenopausal women included those whose status changed from premenopausal to postmenopausal between the 2 screenings.

Other risk factors measured during the second screening were considered covariates in our analysis. Height and weight were measured on the date of screening and used to calculate the BMI. According to the Asia-Pacific classification, BMI was categorized into the following groups [22]: underweight (BMI < 18.5 kg/m^2^), normal (18.5 to < 23.0 kg/m^2^), overweight (23.0 to < 25.0 kg/m^2^), and obese (≥ 25.0 kg/m^2^).

All participants in the national health screening were asked to complete a self-report questionnaire to assess their health behaviors and conditions before the screening. We included the following variables as covariates in our analysis: age at screening, age at menarche (< 15, 15-16, and ≥ 17 years, and missing), child delivery (0, 1, ≥ 2, and missing), duration of breastfeeding (never, < 1 and ≥ 1 year, and missing), oral contraceptive use (never, ever, and missing), family history of breast cancer in first-degree relatives (yes and no), vigorous or moderate physical activity per week (once or more per week, none, or missing), smoking status (never, ever, or missing), alcohol consumption (none, once and ≥ 2 times per week, and missing), and breast density (4 levels). For perimenopausal and postmenopausal women, we further adjusted for age at menopause (< 45, 45-52, and ≥ 53 years, and missing) and hormone replacement therapy for postmenopausal women (never, < 5 and ≥ 5 years, and missing). Only age at screening was expressed as a continuous variable, and the rest of the variables were expressed as categorical variables.

### Statistical analysis

Descriptive statistics of the baseline characteristics of the participants, MetS status at the first and second screenings, and changes in MetS according to menopausal status are presented. To quantify the association between changes in MetS and cancer development, we performed Cox proportional hazards regression analysis to estimate hazard ratios (HRs) and 95% confidence intervals (CIs). The time-to-event period was counted in days from the date of the second screening to the date of the event (breast or endometrial cancer diagnosis), the development of other types of cancer, death, or the end of the study period. The risk of cancer was calculated with adjustment for other covariates. Missing covariate values were treated as dummy variables (separate categories).

We assessed the risk of cancer in the different MetS-change groups compared to the MetS-free group. The MetS-change variable, with 4 values corresponding to the four MetS-change groups, was included in a regression model, and the MetS-free group was used as the reference. We assessed the risk of cancer in the total population and each menopausal group. Three models were used in this study. The first was adjusted for age at the time of screening. The second and third covariates were additionally adjusted for other covariates, with and without adjustment for BMI. Statistical analyses were performed using SAS version 9.4 (SAS Institute Inc., Cary, NC, USA).

### Ethics statement

This study was approved by the Institutional Review Board of Hanyang University College of Medicine (approval No. HYUIRB-202106-003-1). The NHIS database approved the use of the National Health Insurance Service-National Health Information Database (NHIS-NHID). In this study, all members of the screened population agreed to transfer their screening results to the NHIS-NHID. The NHIS database was constructed for individual identification after anonymization.

## RESULTS

### Characteristics of the study population

At the time of screening, the mean± standard deviation (SD) age of 3,031,980 women was 55.5 ± 10.1 years. Among them, 788,926 (26.0%), 229,951 (7.6%), and 2,013,103 (66.4%) women were in the premenopausal, perimenopausal, and postmenopausal phases, respectively (Table 1). The proportions of overweight and obese women in the total population were 25.7% and 33.6%, respectively. After a median follow-up of 8.8 years (interquartile range [IQR], 8.3 to 9.4), 39,148 breast cancer cases and 4,298 endometrial cancer cases were detected.

#### MetS and its change between the 2 screenings

At the first screening, the proportion of women with MetS (≥ 3 criteria) was 26.1% in the total population and was higher in postmenopausal women (32.9%) than in perimenopausal or premenopausal women (17.5 and 11.4%, respectively) (Supplementary Material 1). Between the 2 screenings, 62.7% of women were MetS-free, 10.8% recovered from MetS, 11.1% developed MetS, and 15.4% had persistent MetS. The highest percentage of MetS persistence was found among postmenopausal women (19.8%), followed by perimenopausal (9.5%) and premenopausal women (5.7%). Characteristics according to changes in MetS are described in Table 2. Women with persistent MetS had the highest mean± SD age at 61.3± 9.2 years, and women who were MetS-free had the lowest mean± SD age at 53.4± 10.0 years.

### Association between changes in metabolic syndrome and the risk of breast cancer

In postmenopausal women, those in the MetS-recovered, MetS-developed, or MetS-persistent groups had a higher risk of breast cancer than those in the MetS-free group, with adjusted HRs of 1.19 (95% CI, 1.14 to 1.25), 1.19 (95% CI, 1.14 to 1.24), and 1.33 (95% CI, 1.28 to 1.38), respectively (Table 3). After additionally adjusting for BMI, the HR decreased (1.09, 1.06, and 1.12, respectively), but remained statistically significant. No significant association was found between changes in MetS and the risk of breast cancer after adjusting for BMI in premenopausal and perimenopausal women. Consistent findings was observed in subgroup according to age group (Supplementary Material 2). A subgroup analysis revealed that both non-obese and obese women had an increased risk of postmenopausal breast cancer associated with changes in MetS (Supplementary Material 3).

### Association between changes in metabolic syndrome and risk of endometrial cancer

Changes in MetS were also associated with the risk for endometrial cancer (Table 4). Before adjusting for BMI, women in the MetS-recovered, MetS-developed, and MetS-persistent groups had a 1.36-fold (95% CI, 1.23 to 1.50), 1.28-fold (95% CI, 1.16 to 1.41), and 1.65-fold (95% CI, 1.51 to 1.79) increased risk of endometrial cancer compared to MetS-free women. After adjusting for BMI, the strength of the association decreased, but remained statistically significant, with adjusted HRs of 1.14, 1.04, and 1.22, respectively. Persistent MetS was associated with an increased risk of endometrial cancer in premenopausal and perimenopausal women, with adjusted HRs of 1.41 (95% CI, 1.17 to 1.70) and 1.59 (95% CI, 1.19 to 2.12). Postmenopausal women in the MetS-recovered, MetS-developed, and MetS-persistent groups had a higher risk of endometrial cancer than the MetS-free group, with adjusted HRs of 1.24 (95% CI, 1.09 to 1.40), 1.14 (95% CI, 1.00 to 1.30), and 1.47 (95% CI, 1.32 to 1.63). When stratified by obesity status at the second screening, in non-obese women, a significantly elevated risk of endometrial cancer was found only in postmenopausal women in the MetS-developed and MetS-persistent groups compared with the MetS-free group, with HRs of 1.22 (95% CI 1.02 to 1.45) and 1.34 (95% CI 1.12 to 1.60), respectively (Table 5). Meanwhile, obesity in the MetS-persistent group was associated with an elevated risk of endometrial cancer in premenopausal, perimenopausal, and postmenopausal women (adjusted HRs of 1.49, 1.76, and 1.46, respectively).

## DISCUSSION

This study reports an elevated risk of breast cancer in postmenopausal women who recovered from MetS, developed MetS, and had persistent MetS compared to women who remained free of MetS, after adjusting for BMI and other covariates, but not in premenopausal or perimenopausal women. The risk of endometrial cancer was found to be higher in MetS-persistent and MetS-recovered premenopausal and postmenopausal women. An increased risk of endometrial cancer was also observed in perimenopausal women with MetS-persistent status. When stratifying by obesity status, consistent results were observed in obese women. In non-obese women, an increased endometrial cancer risk associated with MetS development and persistence was only observed in postmenopausal women.

The increased risk of breast cancer in postmenopausal women with MetS is similar to that reported in previous studies [13,23]. A pooled analysis from 3 previous cohort studies reported no significant relationship between MetS and the risk of breast cancer in premenopausal women (risk ratio [RR], 0.91; 95% CI, 0.64 to 1.29) and a 2-fold increased risk in postmenopausal women (RR, 2.01; 95% CI, 1.55 to 2.60) [13]. Regarding the association with changes in MetS, a previous study in Korean women [24] also found an increased risk of breast cancer in women who recovered from MetS, developed MetS, or had persistent MetS. However, a limitation of the previous study [24] was the short follow-up period for cancer development, which was improved in our study (8.6 vs. 6.4 years). In addition, a previous study [24] did not adjust for other important factors of breast cancer, such as reproductive factors, and did not consider the impact of BMI in the analysis. While BMI is an indicator of general obesity, central obesity is an important component of MetS. Although general obesity (assessed by BMI) and MetS are closely related to each other [25], general obesity and the presence of MetS do not always co-occur, suggesting the possibility of an independent role for general obesity and MetS in health status. To date, only a few studies have examined the relative contributions of general obesity, central obesity (assessed by WC), and MetS to the risk of breast cancer [26-28]. Our findings are consistent with the results of the Sister Study [28], which found that postmenopausal women who were metabolically unhealthy, including recovered, developed, and persistent groups, had an elevated risk of breast cancer regardless of their BMI. In this study, postmenopausal women whose MetS status had changed were at an increased risk of breast cancer with similar HRs (1.09 and 1.06 for the MetS-recovered and developed groups, respectively). Based on these results, the effect of short-term changes in MetS on the risk of breast cancer is minimal. Considering the higher HR of the MetS-persistent group compared with other MetS statuses, long-term exposure to the unhealthy status of MetS would affect the future risk of breast cancer in postmenopausal women. More longitudinal studies are needed to determine the duration of MetS, its phenotype, and the future risk of breast cancer.

The association between MetS and the risk of endometrial cancer has been well documented [8,29]. An interesting finding of this study is that the association between changes in MetS and the risk of endometrial cancer differed according to general obesity and menopausal status. Associations between MetS recovery, MetS persistence, and the risk of endometrial cancer were observed in obese women, irrespective of menopausal status. Several studies have found an association between MetS and an increased risk of endometrial cancer in obese women [12,30,31]. Even though we did not know participants’ MetS status before the first screening, the total exposure duration of MetS in MetS-recovered obese women was expected to be longer than that in women with newly developed MetS. To confirm this hypothesis, prospective studies on the development of MetS and endometrial cancer in young women need to be conducted. In this study, the association between endometrial cancer and MetS persistence was observed only in postmenopausal, non-obese women. The increased risk of endometrial cancer in non-obese women who developed MetS between the first and second screening could be due to weight gain within the normal range (Supplementary Material 4), followed by worsening metabolic health. This might suggest a population subgroup that is susceptible to disease risk with an increased weight even within the normal range, followed by MetS development and further risk of endometrial cancer. Non-obese women with metabolic abnormalities showed lower circulating insulin levels than obese women [32]. Despite the general non-association between MetS and endometrial cancer in non-obese women [12,30,31], postmenopausal women who developed MetS or had persistent MetS were at a higher risk of endometrial cancer than premenopausal or perimenopausal women. The increased disease risk in postmenopausal women with MetS compared to women without MetS, including cancer, cardiovascular diseases, and other chronic diseases, has been well identified. However, few studies have explored this issue in premenopausal women. Considering the very low incidence of endometrial cancer in young women [33] and the increased incidence of endometrial cancer with increasing age [34,35], non-obese premenopausal and perimenopausal women would have too low a risk of developing endometrial cancer during the follow-up period to obtain meaningful results.

Shared etiologies and risk factors between breast cancer and endometrial cancer have been identified [36,37], as have shared physiological mechanisms underlying the association of MetS with breast and endometrial cancer [9,38,39]. Obesity-related insulin resistance, hyperinsulinemia, impaired glucose tolerance, dyslipidemia, and visceral obesity are some of the biological mechanisms that contribute to the impact of MetS on the risk of endometrial cancer [9,39]. Obesity-related metabolic factors, including serum TG levels, were shown to be associated with increased endometrial cancer risk [40]; however, further adjustment for BMI tended to attenuate the association, which was likewise observed in our results (Supplementary Material 5). Insulin resistance, inflammation, and estrogen from adipose tissue have been suggested to be the main physiological mechanisms involved in the increased risk of breast cancer related to MetS in postmenopausal women [38]. The different associations between changes in MetS and breast and endometrial cancer based on menopausal status and obesity may reflect the different age-specific incidence rates of those cancers [33,41].

This study has several limitations. First, the median follow-up time was less than 9 years, which was relatively short and might be insufficient to assess the effect of MetS on the risk of cancer, as a relatively slowly developing disease. Second, our database may have been subjected to selection bias because women who regularly undergo cancer screening may be a particularly health-conscious segment of the population. Third, data on MetS status before the first screening were not available and were therefore not considered in the current findings. This left-truncated feature of the database might explain some of the higher HRs observed in women who recovered from MetS relative to those observed in the MetS-free group. Finally, this study assessed changes in MetS using only 2 biennial cancer screenings. The short period between the 2 measurements and the limited number of MetS assessments (only 2 measurements) may not have fully captured the trajectory of MetS changes over time. As MetS is a chronic condition that can develop over many years, there may have been some misclassification of participants’ MetS statuses between the 2 screenings owing to transient changes in the MetS criteria. This misclassification of MetS status may have led to an underestimation or overestimation of the impact of MetS changes on cancer risk. Thus, future studies with different approaches for assessing MetS changes—either with longer gaps between 2 assessments or more total MetS assessments—may be helpful for overcoming our study’s limitations.

While increased breast and endometrial cancer risks associated with MetS have been previously reported [9,13], those studies used only single measurements of MetS. In this study, our results were based on changes in MetS. Our findings suggest that alterations in MetS status and sustained MetS, especially in postmenopausal women, are associated with increased risks of breast and endometrial cancer. Thus, these results imply that improvements in MetS status may help to reduce the risk of developing these cancers. Transitions in MetS status may be achieved through lifestyle modifications such as smoking cessation, increased physical activity, diet, and maintaining a healthy weight.

Despite these limitations, our study has several strengths. First, this study included a very large, women, population-based database with a longitudinal assessment of MetS status and an accurate assessment of cancer cases. In addition, a wide range of well-established confounding factors associated with breast cancer and endometrial cancer were adjusted for, such as reproductive factors and family history of cancer. We also excluded prevalent cancer cases that developed within 6 months of the last assessment of MetS to avoid the potential inverse causality between changes in MetS and cancer development.

In conclusion, this cohort study found that changes in MetS were associated with an increased risk of breast and endometrial cancer in Korean women aged ≥ 40 years, with the highest risk in those who had persistent MetS. MetS-persistent status in postmenopausal women was associated with increased breast cancer risk. A higher risk of endometrial cancer was found in obese women who recovered from MetS or had persistent MetS, whereas in non-obese women, MetS only showed significant associations in the postmenopausal subgroup. Our findings add more evidence to support the idea that not only MetS itself, but also its changes, are important etiologic factors for breast and endometrial cancer. Hence, nutritional and lifestyle modifications to alleviate MetS may help reduce the risk of these cancers.

## DATA AVAILABILITY

The data that support the findings of this study are available on the website of the National Health Insurance Sharing Service (https://nhiss.nhis.or.kr/). We accessed the database after submitting the study protocol, the IRB approval document, and the reviewed request form by the committee. Further information is available from the corresponding author upon request.

## Figures and Tables

**Figure f3-epih-45-e2023049:**
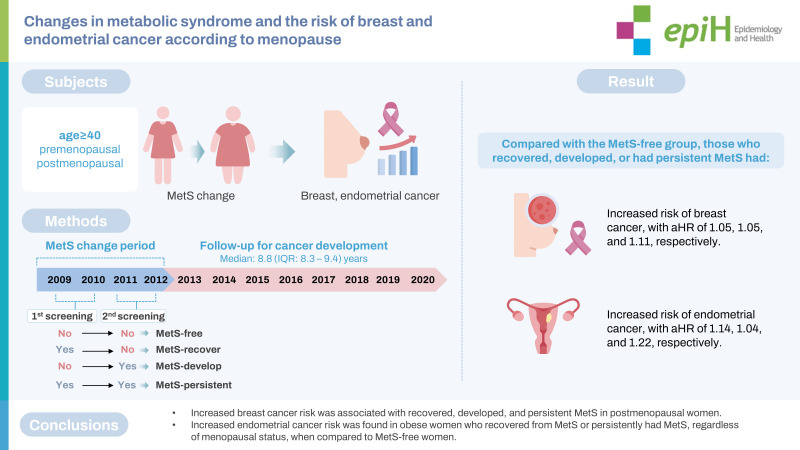


**Table 1.** Descriptive statistics of the study population according to menopausal status at the second screening

Characteristics		Total (n=3,031,980)	Premenopausal (n=788,926)	Perimenopausal (n=229,951)	Postmenopausal (n=2,013,103)
Age, mean±SD (yr)		55.5±10.1	44.1±3.5	49.8±4.4	60.6±8.0
Age group (yr)					
	<50	701,683 (23.1)	616,392 (78.1)	45,696 (19.9)	39,595 (2.0)
	50-59	1,063,138 (35.1)	171,923 (21.8)	174,700 (76.0)	716,515 (35.6)
	60-69	793,473 (26.2)	500 (0.1)	6,943 (3.0)	786,030 (39.1)
	≥70	473,686 (15.6)	111 (0.0)	2,612 (1.1)	470,963 (23.4)
BMI status (kg/m^2^)					
	<18.5	62,832 (2.1)	21,819 (2.8)	4,669 (2.0)	36,344 (1.8)
	18.5 to <23.0	1,172,660 (38.7)	389,117 (49.3)	99,094 (43.1)	684,449 (34.0)
	23.0 to <25.0	778,237 (25.7)	182,802 (23.2)	58,368 (25.4)	537,067 (26.7)
	≥25.0	1,018,251 (33.6)	195,188 (24.7)	67,820 (29.5)	755,243 (37.5)
Age at menarche (yr)					
	<15	666,017 (22.0)	311,503 (39.5)	59,099 (25.7)	295,415 (14.7)
	16-17	1,243,074 (41.0)	349,921 (44.4)	104,468 (45.4)	788,685 (39.2)
	>17	1,102,650 (36.4)	121,902 (15.5)	64,418 (28.0)	916,330 (45.5)
	Missing	20,239 (0.7)	5,600 (0.7)	1,966 (0.9)	12,673 (0.6)
Children delivered					
	None	104,100 (3.4)	38,758 (4.9)	8,594 (3.7)	56,748 (2.8)
	One	267,986 (8.8)	108,240 (13.7)	26,885 (11.7)	132,861 (6.6)
	Two or more	2,655,750 (87.6)	640,496 (81.2)	193,958 (84.4)	1,821,296 (90.5)
	Missing	4,144 (0.1)	1,432 (0.2)	514 (0.2)	2,198 (0.1)
Duration of breastfeeding (yr)					
	Never	295,438 (9.7)	136,241 (17.3)	28,527 (12.4)	130,670 (6.5)
	<1	977,449 (32.2)	410,321 (52.0)	93,736 (40.8)	473,392 (23.5)
	≥1	1,703,524 (56.2)	221,951 (28.1)	102,801 (44.7)	1,378,772 (68.5)
	Missing	55,569 (1.8)	20,413 (2.6)	4,887 (2.1)	30,269 (1.5)
Oral contraceptive use					
	Never	2,450,978 (80.8)	660,712 (83.8)	190,616 (82.9)	1,599,650 (79.5)
	Ever	452,767 (14.9)	100,656 (12.8)	29,321 (12.8)	322,790 (16.0)
	Missing	128,235 (4.2)	27,558 (3.5)	10,014 (4.4)	90,663 (4.5)
Family history of breast cancer in first-degree relatives					
	No	2,971,199 (98.0)	769,864 (97.6)	224,661 (97.7)	1,976,674 (98.2)
	Yes	60,781 (2.0)	19,062 (2.4)	5,290 (2.3)	36,429 (1.8)
Vigorous or moderate physical activity per week					
	None	2,617,856 (86.3)	638,661 (81)	192,902 (83.9)	1,786,293 (88.7)
	Once or more per week	411,626 (13.6)	149,677 (19)	36,826 (16.0)	225,123 (11.2)
	Missing	2,498 (0.1)	588 (0.1)	223 (0.1)	1,687 (0.1)
Smoking status					
	Never	2,922,826 (96.4)	751,971 (95.3)	219,978 (95.7)	1,950,877 (96.9)
	Ever	107,003 (3.5)	36,360 (4.6)	9,745 (4.2)	60,898 (3.0)
	Missing	2,151 (0.1)	595 (0.1)	228 (0.1)	1,328 (0.1)
Alcohol consumption					
	None	2,497,036 (82.4)	550,290 (69.8)	176,034 (76.6)	1,770,712 (88.0)
	Once per week	319,933 (10.6)	145,811 (18.5)	31,306 (13.6)	142,816 (7.1)
	Twice or more per week	212,851 (7.0)	92,317 (11.7)	22,425 (9.8)	98,109 (4.9)
	Missing	2,160 (0.1)	508 (0.1)	186 (0.1)	1,466 (0.1)
Age at menopause (yr)					
	<50	1,709,977 (56.4)	-	107,091 (46.6)	813,960 (40.4)
	50 to <55	1,083,546 (35.7)	-	109,742 (47.7)	973,804 (48.4)
	≥55	238,457 (7.9)	-	13,118 (5.7)	225,339 (11.2)
Hormone replacement therapy among postmenopausal women					
	No	1,612,539 (53.2)	-	172,285 (74.9)	1,440,254 (71.5)
	Yes	330,332 (10.9)	-	28,694 (12.5)	301,638 (15.0)
	Missing or NA	1,089,109 (35.9)	788,926 (100)	28,972 (12.6)	271,211 (13.5)

Values are presented as number (%). SD, standard deviation; BMI, body mass index; NA, not available.

**Table 2.** Descriptive statistics of the study population according to MetS change status

Characteristics		Free (n=1,901,972)	Recovered (n=326,705)	Developed (n=337,858)	Persistent (n=465,445)
Age, mean±SD (yr)		53.4±10.0	59.6±9.6	58.6±9.7	61.3±9.2
Total cholesterol (mg/dL)		199.8 (36.0)	203 (39.2)	206.6 (41.1)	205.3 (42.5)
HDL (mg/dL)		61 (22.6)	56.1 (25.9)	50.3 (17.1)	47.8 (14.2)
LDL (mg/dL)		119.8 (36.0)	123.2 (39.3)	123.6 (40.8)	120.8 (42.8)
Fasting glucose level (mg/dL)		92.5 (14.6)	99.2 (22.3)	105.2 (23.5)	113.9 (30.9)
Systolic blood pressure (mmHg)		118.2 (14.2)	124.5 (14.4)	131.5 (14.4)	134 (15.0)
Diastolic blood pressure (mmHg)		73.3 (9.4)	76.4 (9.2)	80.1 (9.5)	80.9 (9.8)
Age group (yr)					
	<50	601,594 (31.6)	38,388 (11.8)	45,408 (13.4)	37,096 (8.0)
	50-59	667,022 (35.1)	90,334 (27.7)	103,587 (30.7)	110,869 (23.8)
	60-69	399,414 (21.0)	115,425 (35.3)	111,966 (33.1)	173,239 (37.2)
	≥70	233,943 (12.3)	82,558 (25.3)	76,896 (22.8)	144,242 (31.0)
BMI status (kg/m^2^)					
	<18.5	59,151 (3.1)	2,450 (0.8)	1,993 (0.6)	977 (0.2)
	18.5-<23.0	905,339 (47.6)	79,226 (24.3)	68,585 (20.3)	56,412 (12.1)
	23.0-<25.0	450,197 (23.7)	87,198 (26.7)	92,033 (27.2)	105,051 (22.6)
	≥25.0	371,075 (19.5)	139,895 (42.8)	175,213 (51.9)	302,958 (65.1)
	Missing				
Age at menarche (yr)					
	<15	455,522 (24.0)	51,815 (15.9)	56,084 (16.6)	67,071 (14.4)
	16-17	789,509 (41.5)	125,847 (38.5)	130,379 (38.6)	174,263 (37.4)
	>17	632,786 (33.3)	144,600 (44.3)	147,475 (43.7)	218,387 (46.9)
	Missing	24,155 (1.3)	4,443 (1.4)	3,919 (1.2)	5,725 (1.2)
Children delivered					
	One	77,600 (4.1)	8,560 (2.6)	10,102 (3.0)	11,310 (2.4)
	Two or more	195,142 (10.3)	21,759 (6.7)	23,346 (6.9)	25,693 (5.5)
	None	1,616,486 (85.0)	294,329 (90.1)	302,417 (89.5)	425,836 (91.5)
	Missing	12,743 (0.7)	2,058 (0.6)	1,993 (0.6)	2,606 (0.6)
Duration of breastfeeding (yr)					
	No	211,689 (11.1)	22,216 (6.8)	23,853 (7.1)	26,437 (5.7)
	<1	679,955 (35.8)	76,384 (23.4)	81,221 (24.0)	91,600 (19.7)
	≥1	961,827 (50.6)	221,833 (67.9)	225,588 (66.8)	338,937 (72.8)
	Missing	48,500 (2.6)	6,273 (1.9)	7,196 (2.1)	8,472 (1.8)
Oral contraceptive use					
	Never	1,545,733 (81.3)	258,881 (79.2)	269,239 (79.7)	366,352 (78.7)
	Ever	268,558 (14.1)	51,260 (15.7)	52,300 (15.5)	76,798 (16.5)
	Missing	87,871 (4.6)	16,564 (5.1)	16,319 (4.8)	22,294 (4.8)
Family history of breast cancer					
	No	1,865,074 (98.1)	321,772 (98.5)	332,655 (98.5)	458,882 (98.6)
	Yes	36,898 (1.9)	4,933 (1.5)	5,203 (1.5)	6,563 (1.4)
Vigorous/moderate physical activity					
	None	1,521,007 (80.0)	273,485 (83.7)	297,687 (88.1)	416,666 (89.5)
	Once or more per week	262,853 (13.8)	35,088 (10.7)	39,833 (11.8)	48,360 (10.4)
	Missing	118,112 (6.2)	18,132 (5.6)	338 (0.1)	419 (0.1)
Smoking					
	Never	1,718,622 (90.4)	297,498 (91.1)	324,276 (96)	448,922 (96.5)
	Ever	65,618 (3.5)	11,075 (3.4)	13,278 (3.9)	16,151 (3.5)
	Missing	117,922 (6.2)	18,132 (5.6)	304 (0.1)	372 (0.1)
Alcohol consumption					
	None	1,412,975 (74.3)	264,958 (81.1)	287,754 (85.2)	412,058 (88.5)
	Once per week	226,525 (11.9)	25,614 (7.8)	28,481 (8.4)	30,347 (6.5)
	Twice or more per week	144,740 (7.6)	17,969 (5.5)	21,386 (6.3)	22,667 (4.9)
	Missing	117,732 (6.2)	18,165 (5.6)	237 (0.1)	373 (0.1)
Age at menopause (yr)					
	<50	1,191,776 (62.7)	162,111 (49.6)	170,449 (50.5)	216,199 (46.5)
	50-<55	595,507 (31.3)	131,825 (40.4)	134,873 (39.9)	196,092 (42.1)
	≥55	114,689 (6.0)	32,769 (10.0)	32,536 (9.6)	53,153 (11.4)
Hormone replacement therapy among postmenopausal women					
	No	888,601 (46.7)	212,914 (65.2)	216,567 (64.1)	329,163 (70.7)
	Yes	184,872 (9.7)	30,449 (9.3)	31,421 (9.3)	37,655 (8.1)
	Missing	828,499 (43.6)	83,342 (25.5)	89,870 (26.6)	98,628 (21.2)

Values are presented as number (%). MetS, metabolic syndrome; SD, standard deviation; HDL, high-density lipoprotein; LDL, low-density lipoprotein; BMI, body mass index.

**Table 3.** Association between changes in metabolic syndrome (MetS) and the risk of breast cancer development^1^

Changes in MetS		No. of cases	Person-years	Model 1^1^	Model 2^2^	Model 3^3^
Total						
	Free	25,919	16,428,716	1.00 (reference)	1.00 (reference)	1.00 (reference)
	Recovered	3,772	2,804,089	1.05 (1.02, 1.09)	1.13 (1.10, 1.18)	1.05 (1.02, 1.09)
	Developed	4,046	2,897,548	1.07 (1.03, 1.10)	1.15 (1.11, 1.19)	1.05 (1.02, 1.09)
	Persistent	5,411	3,964,028	1.14 (1.11, 1.18)	1.26 (1.22, 1.30)	1.11 (1.00, 1.14)
Premenopausal						
	Free	12,167	5,594,895	1.00 (reference)	1.00 (reference)	1.00 (reference)
	Recovered	771	384,776	0.93 (0.87, 1.00)	1.00 (0.93, 1.08)	0.97 (0.90, 1.04)
	Developed	983	449,817	1.02 (0.95, 1.09)	1.09 (1.02, 1.16)	1.05 (0.98, 1.12)
	Persistent	817	386,732	0.99 (0.92, 1.06)	1.09 (1.01, 1.17)	1.03 (0.95, 1.11)
Perimenopausal						
	Free	2,399	1,450,028	1.00 (reference)	1.00 (reference)	1.00 (reference)
	Recovered	286	159,890	1.10 (0.97, 1.24)	1.18 (1.04, 1.33)	1.12 (0.99, 1.27)
	Developed	331	191,356	1.06 (0.94, 1.19)	1.14 (1.02, 1.28)	1.07 (0.95, 1.21)
	Persistent	341	187,006	1.12 (1.00, 1.26)	1.25 (1.11, 1.40)	1.13 (1.00, 1.28)
Postmenopausal						
	Free	11,353	9,383,793	1.00 (reference)	1.00 (reference)	1.00 (reference)
	Recovered	2,715	2,259,422	1.11 (1.07, 1.16)	1.19 (1.14, 1.25)	1.09 (1.04, 1.14)
	Developed	2,732	2,256,375	1.10 (1.06, 1.15)	1.19 (1.14, 1.24)	1.06 (1.02, 1.11)
	Persistent	4,253	3,390,290	1.21 (1.16, 1.25)	1.33 (1.28, 1.38)	1.12 (1.08, 1.16)

Values are presented as hazard ratio (95% confidence interval).

1 Model 1 was adjusted for age at screening.

2 Model 2 was adjusted for age at screening, age at menarche, child delivery, breastfeeding, oral contraceptive use, family history of breast cancer, vigorous or moderate physical activity, smoking status, alcohol consumption, and breast density; For perimenopausal and postmenopausal women, the model was additionally adjusted for age at menopause and the use of hormone replacement therapy.

3 Model 3 was adjusted for covariates similar to model 2, with the addition of body mass index.

**Table 4.** Association between changes in metabolic syndrome (MetS) and the risk of endometrial cancer development

Changes in MetS		No. of cases	Person-years	Model 1^1^	Model 2^2^	Model 3^3^
Total						
	Free	2,568	16,428,716	1.00 (reference)	1.00 (reference)	1.00 (reference)
	Recovered	482	2,804,089	1.33 (1.20, 1.46)	1.36 (1.23, 1.50)	1.14 (1.03, 1.27)
	Developed	480	2,897,548	1.25 (1.13, 1.38)	1.28 (1.16, 1.41)	1.04 (0.94, 1.15)
	Persistent	768	3,964,028	1.58 (1.45, 1.72)	1.65 (1.51, 1.79)	1.22 (1.11, 1.34)
Premenopausal						
	Free	1,124	5,594,895	1.00 (reference)	1.00 (reference)	1.00 (reference)
	Recovered	118	384,776	1.43 (1.18, 1.73)	1.43 (1.18, 1.73)	1.30 (1.07, 1.57)
	Developed	126	449,817	1.31 (1.09, 1.57)	1.31 (1.09, 1.58)	1.17 (0.97, 1.42)
	Persistent	138	386,732	1.63 (1.36, 1.94)	1.61 (1.34, 1.92)	1.41 (1.17, 1.70)
Perimenopausal						
	Free	289	1,450,028	1.00 (reference)	1.00 (reference)	1.00 (reference)
	Recovered	43	159,890	1.31 (0.95, 1.80)	1.36 (0.98, 1.88)	1.27 (0.92, 1.77)
	Developed	48	191,356	1.23 (0.90, 1.67)	1.29 (0.95, 1.75)	1.19 (0.87, 1.63)
	Persistent	64	187,006	1.64 (1.25, 2.16)	1.75 (1.32, 2.31)	1.59 (1.19, 2.12)
Postmenopausal						
	Free	1,155	9,383,793	1.00 (reference)	1.00 (reference)	1.00 (reference)
	Recovered	321	2,259,422	1.30 (1.140, 1.47)	1.36 (1.20, 1.54)	1.24 (1.09, 1.40)
	Developed	306	2,256,375	1.22 (1.07, 1.39)	1.28 (1.13, 1.46)	1.14 (1.00, 1.30)
	Persistent	566	3,390,290	1.59 (1.43, 1.76)	1.70 (1.53, 1.89)	1.47 (1.32, 1.63)

Values are presented as hazard ratio (95% confidence interval).

1 Model 1 was adjusted for age at screening.

2 Model 2 was adjusted for age at screening, age at menarche, child delivery, breastfeeding, oral contraceptive use, family history of breast cancer, vigorous or moderate physical activity, smoking status, alcohol consumption, and breast density; For perimenopausal and postmenopausal women, the model was additionally adjusted for age at menopause and the use of hormone replacement therapy.

3 Model 3 was adjusted for covariates similar to model 2, with the addition of body mass index.

**Table 5.** Association between changes in MetS and the risk of endometrial cancer in obese and non-obese women

Changes in MetS			No. of cases	Person-years	Model 1^1^	Model 2^2^
Non-obese (BMI <25.0 kg/m^2^)						
	Total					
		Free	1,950	13,129,559	1.00 (reference)	1.00 (reference)
		Recovered	180	1,376,020	1.05 (0.90, 1.23)	1.08 (0.92, 1.26)
		Developed	229	1,538,282	1.17 (1.02, 1.34)	1.20 (1.04, 1.38)
		Persistent	180	1,322,467	1.17 (1.00, 1.37)	1.22 (1.04, 1.43)
	Premenopausal					
		Free	899	4,727,718	1.00 (reference)	1.00 (reference)
		Recovered	40	171,897	1.11 (0.81, 1.53)	1.13 (0.83, 1.56)
		Developed	59	229,162	1.24 (0.95, 1.62)	1.26 (0.97, 1.65)
		Persistent	23	98,465	1.07 (0.70, 1.61)	1.09 (0.72, 1.65)
	Perimenopausal					
		Free	229	1,182,722	1.00 (reference)	1.00 (reference)
		Recovered	14	74,060	0.91 (0.53, 1.56)	0.94 (0.55, 1.62)
		Developed	24	100,176	1.18 (0.77, 1.79)	1.23 (0.81, 1.88)
		Persistent	13	54,455	1.09 (0.62, 1.93)	1.16 (0.66, 2.05)
	Postmenopausal					
		Free	822	7,219,118	1.00 (reference)	1.00 (reference)
		Recovered	126	1,130,063	1.10 (0.91, 1.32)	1.14 (0.94, 1.37)
		Developed	146	1,208,945	1.17 (0.98, 1.40)	1.22 (1.02, 1.45)
		Persistent	144	1,169,547	1.26 (1.05, 1.51)	1.34 (1.12, 1.60)
Obese (BMI ≥25.0 kg/m^2^)						
	Total					
		Free	618	3,299,157	1.00 (reference)	1.00 (reference)
		Recovered	302	1,428,069	1.30 (1.13, 1.49)	1.30 (1.13, 1.49)
		Developed	251	1,359,266	1.11 (0.96, 1.29)	1.11 (0.96, 1.29)
		Persistent	588	2,641,561	1.47 (1.30, 1.65)	1.47 (1.31, 1.65)
	Premenopausal					
		Free	225	867,177	1.00 (reference)	1.00 (reference)
		Recovered	78	212,879	1.39 (1.08, 1.80)	1.39 (1.07, 1.79)
		Developed	67	220,655	1.16 (0.88, 1.52)	1.15 (0.88, 1.51)
		Persistent	115	288,267	1.50 (1.20, 1.88)	1.49 (1.19, 1.87)
	Perimenopausal					
		Free	60	267,306	1.00 (reference)	1.00 (reference)
		Recovered	29	85,830	1.51 (0.97, 2.35)	1.53 (0.98, 2.39)
		Developed	24	91,180	1.18 (0.73, 1.89)	1.19 (0.74, 1.92)
		Persistent	51	132,551	1.72 (1.18, 2.51)	1.76 (1.21, 2.57)
	Postmenopausal					
		Free	333	2,164,675	1.00 (reference)	1.00 (reference)
		Recovered	195	1,129,359	1.23 (1.03, 1.47)	1.25 (1.05, 1.49)
		Developed	160	1,047,430	1.07 (0.89, 1.30)	1.10 (0.91, 1.32)
		Persistent	422	2,220,743	1.42 (1.22, 1.64)	1.46 (1.26, 1.69)

Values are presented as hazard ratio (95% confidence interval). MetS, metabolic syndrome; BMI, body mass index.

1 Model 1 was adjusted for age at screening, age at menarche, child delivery, breastfeeding, oral contraceptive use, family history of breast cancer, vigorous or moderate physical activity, smoking status, alcohol consumption, and breast density; For perimenopausal and postmenopausal women, the model was additionally adjusted for age at menopause and the use of hormone replacement therapy.

2 Model 2 was adjusted for covariates similar to model 1, with the addition of BMI.

## SUPPLEMENTARY MATERIALS

Supplementary Material 1. MetS at baseline, follow-up, and changes in MetS between the two screenings

Supplementary Material 2 Association between changes in MetS and the risk of breast/endometrial cancers in premenopausal women according to age at diagnosis (<50 and ≥50 years)

Supplementary Material 3 Association between changes in MetS and the risk of breast cancer in obese and non-obese women

Supplementary Material 4 Mean weight change from first to second screening in obese and non-obese women based on changes in MetS

Supplementary Material 5 Association between changes in MetS and the risk of breast, endometrial cancer by change in MetS components
