# Supplementary material for: Changes in metabolic syndrome and the risk of breast and endometrial cancer according to menopause in Korean women
Source: Epidemiol Health. 2023 May 1;45:e2023049. doi: 10.4178/epih.e2023049 (PMC10593591; doi:10.4178/epih.e2023049)
Supplement: Supplementary Material 1. — MetS at baseline, follow-up, and changes in MetS between the two screenings [file epih-45-e2023049-Supplementary-1.docx]

**Supplementary Materials**

**Supplementary Material 1. MetS at baseline, follow-up, and changes in MetS between the two screenings**

| **MetS** | **Total** | **Premenopausal** | **Perimenopausal** | **Postmenopausal** |
| --- | --- | --- | --- | --- |
|  | **n (%)** | **n (%)** | **n (%)** | **n (%)** |
|  | **n = 3,031,980** | **n = 788,926** | **n = 229,951** | **n = 2,013,103** |
| MetS score at the first screening | |  |  |  |
| None | 688,913 (22.7) | 311,609 (39.5) | 68,866 (30.0) | 308,438 (15.3) |
| One | 828,895 (27.3) | 251,521 (31.9) | 71,487 (31.1) | 505,887 (25.1) |
| Two | 722,022 (23.8) | 136,286 (17.3) | 49,314 (21.5) | 536,422 (26.7) |
| Three | 490,346 (16.2) | 62,171 (7.9) | 26,966 (11.7) | 401,209 (19.9) |
| Four | 237,694 (7.8) | 22,563 (2.9) | 10,794 (4.7) | 204,337 (10.2) |
| Five | 64,110 (2.1) | 4,776 (0.6) | 2,524 (1.1) | 56,810 (2.8) |
| MetS score at the second screening | |  |  |  |
| None | 675,846 (22.3) | 305,834 (38.8) | 65,567 (28.5) | 304,445 (15.1) |
| One | 822,945 (27.1) | 246,302 (31.2) | 69,803 (30.4) | 506,840 (25.2) |
| Two | 729,886 (24.1) | 139,516 (17.7) | 50,610 (22.0) | 539,760 (26.8) |
| Three | 497,554 (16.4) | 66,580 (8.4) | 28,952 (12.6) | 402,022 (20.0) |
| Four | 240,166 (7.9) | 25,197 (3.2) | 12,125 (5.3) | 202,844 (10.1) |
| Five | 65,583 (2.2) | 5,497 (0.7) | 2,894 (1.3) | 57,192 (2.8) |
| Changes in MetS |  |  |  |  |
| Free | 1,901,972 (62.7) | 647,154 (82) | 167,472 (72.8) | 1,087,346 (54.0) |
| Recovered | 326,705 (10.8) | 44,498 (5.6) | 18,508 (8.1) | 263,699 (13.1) |
| Developed | 337,858 (11.1) | 52,262 (6.6) | 22,195 (9.7) | 263,401 (13.1) |
| Persistent | 465,445 (15.4) | 45,012 (5.7) | 21,776 (9.5) | 398,657 (19.8) |

MetS, metabolic syndrome.
