# Supplementary material for: Changes in metabolic syndrome and the risk of breast and endometrial cancer according to menopause in Korean women
Source: Epidemiol Health. 2023 May 1;45:e2023049. doi: 10.4178/epih.e2023049 (PMC10593591; doi:10.4178/epih.e2023049)
Supplement: Supplementary Material 2 — Association between changes in MetS and the risk of breast/endometrial cancers in premenopausal women according to age at diagnosis (<50 and ≥50 years) [file epih-45-e2023049-Supplementary-2.docx]

**Supplementary Material 2. Association between changes in MetS and the risk of breast/endometrial cancers in premenopausal women according to age at diagnosis (<50 and ≥50 years)**

| **Change in MetS status** | **No. of cases** | **Model 1** | |  | **Model 2** | |
| --- | --- | --- | --- | --- | --- | --- |
|  |  | **HR** | **95% CI** |  | **HR** | **95% CI** |
| **BREAST CANCER** |  |  |  |  |  |  |
| **Age at diagnosis <50** |  |  |  |  |  |  |
| Free | 5705 |  |  |  |  |  |
| Recovered | 313 | 0.96 | (0.85 - 1.09) |  | 1.10 | (0.97 - 1.24) |
| Developed | 410 | 1.06 | (0.95 - 1.19) |  | **1.26** | **(1.12 - 1.41)** |
| Persistent | 310 | 1.03 | (0.91 - 1.16) |  | **1.23** | **(1.09 - 1.40)** |
| **Age at diagnosis ≥50** | |  |  |  |  |  |
| Free | 6462 |  |  |  |  |  |
| Recovered | 458 | 0.96 | (0.86 - 1.07) |  | 0.86 | (0.77 - 0.96) |
| Developed | 573 | 1.06 | (0.96 - 1.17) |  | **1.19** | **(1.08 - 1.31)** |
| Persistent | 507 | 1.03 | (0.93 - 1.14) |  | 0.93 | (0.84 - 1.03) |
| **ENDOMETRIAL CANCER** | |  |  |  |  |  |
| **Age at diagnosis <50** |  |  |  |  |  |  |
| Free | 321 |  |  |  |  |  |
| Recovered | 30 | **1.91** | **(1.31 - 2.79)** |  | 1.36 | (0.93 - 1.98) |
| Developed | 40 | **2.33** | **(1.68 - 3.24)** |  | **1.40** | **(1.01 - 1.95)** |
| Persistent | 46 | **3.48** | **(2.56 - 4.74)** |  | **1.66** | **(1.22 - 2.26)** |
| **Age at diagnosis ≥50** | |  |  |  |  |  |
| Free | 803 |  |  |  |  |  |
| Recovered | 89 | 0.92 | (0.74 - 1.15) |  | 1.05 | (0.84 -1.31) |
| Developed | 86 | **1.75** | **(1.40 - 2.19)** |  | **1.92** | **(1.54 - 2.40)** |
| Persistent | 92 | 0.87 | (0.70 - 1.08) |  | 0.85 | (0.69 - 1.05) |

^a^ Model 1 was adjusted for age at screening, age at menarche, child delivery, breastfeeding, oral contraceptive use, family history of breast cancer, vigorous or moderate physical activity, smoking status, alcohol consumption, and breast density. For perimenopausal and postmenopausal women, the model was additionally adjusted for age at menopause and the use of hormone replacement therapy.

^d^ Model 2 was adjusted for covariates similar to Model 1 and with the addition of BMI.

CI, confidence interval; HR, hazard ratio; BMI, body mass index
