# Supplementary material for: Changes in metabolic syndrome and the risk of breast and endometrial cancer according to menopause in Korean women
Source: Epidemiol Health. 2023 May 1;45:e2023049. doi: 10.4178/epih.e2023049 (PMC10593591; doi:10.4178/epih.e2023049)
Supplement: Supplementary Material 4 — Mean weight change from first to second screening in obese and non-obese women based on changes in MetS [file epih-45-e2023049-Supplementary-4.docx]

**Supplementary Material 4. Mean weight change from first to second screening in obese and non-obese women based on changes in MetS**

| **Changes in MetS** | **Non-obese (BMI <25 kg/m^2^)** | | **Obese (BMI ≥25 kg/m^2^)** | | **p-value** |
| --- | --- | --- | --- | --- | --- |
|  | **Mean** | **SD** | **Mean** | **SD** |  |
| Premenopausal |  |  |  |  |  |
| Free | 0.44 | 2.52 | -0.54 | 3.76 | <.001 |
| Recovered | -0.28 | 2.88 | -1.58 | 4.05 | <.001 |
| Developed | 1.70 | 3.04 | 1.02 | 3.47 | <.001 |
| Persistent | 0.79 | 2.89 | -0.06 | 3.42 | <.001 |
| Perimenopausal |  |  |  |  |  |
| Free | 0.11 | 2.57 | -0.77 | 3.50 | <.001 |
| Recovered | -0.55 | 2.84 | -1.51 | 3.70 | <.001 |
| Developed | 1.16 | 2.97 | 0.37 | 3.32 | <.001 |
| Persistent | 0.37 | 2.92 | -0.44 | 3.32 | <.001 |
| Postmenopausal |  |  |  |  |  |
| Free | -0.03 | 2.59 | -0.89 | 3.27 | <.001 |
| Recovered | -0.71 | 2.88 | -1.52 | 3.42 | <.001 |
| Developed | 0.78 | 2.88 | -0.15 | 3.18 | <.001 |
| Persistent | 0.20 | 2.99 | -0.75 | 3.21 | <.001 |

MetS, metabolic syndrome; BMI, body mass index; SD, standard deviation
